# RUNNING PAST 50: THE FORTITUDE OF THE OLDER RUNNER

**DOI:** 10.1093/geroni/igae098.0297

**Published:** 2024-12-31

**Authors:** James Dowd

**Affiliations:** University of Georgia, Athens, Georgia, United States

## Abstract

Considering the low numbers of physically active older Americans (Elgaddal, Kramarow, Reuben, 2022), those over the age of 50 who belie this generalization are of interest for their commitment to fitness. Despite their regret over their loss of running speed, any comparison with their more sedentary age peers would favor the older runner: they are healthier, fitter, and stronger. The question I address in this paper is why the older runner continues to run. Based on a sample of over 60 interviews conducted from July to December of 2023, I found that it is not simply a matter of striving to be healthy, although that is part of their motivation. As important as health are (1) running has been a habit and consequently a foundational part of their identity; and (2) running brings joy and a sense of profound well-being that older runners experience following a race, almost regardless of their performance. When asked about their motivation, runners acknowledge the positive effects of exercise on their health. But as one 72-year-old runner explained “Well, physical health and conditioning is important. But I wouldn’t say. I mean, that’s a benefit. I wouldn’t say that’s why I run.” The remainder of this paper probes more deeply into what might best be described as the fortitude of the older runner. The data confirm that older runners persist both because their running has become a habitual part of their everyday lives and also because their habit is a source of considerable joy for them.

